# Relationship between lymphocyte-related parameters and the prognosis of patients with lupus nephritis

**DOI:** 10.3389/fimmu.2025.1613483

**Published:** 2025-07-08

**Authors:** Wenyi Qi, Rong Zhu, Xue Bai, Ping Luo, Manyu Luo

**Affiliations:** The Department of Nephropathy, The Second Hospital of Jilin University, Changchun, Jilin, China

**Keywords:** lupus nephritis, platelet to lymphocyte ratio, neutrophil to lymphocyte ratio, monocyte to lymphocyte ratio, prognosis, retrospective cohort study

## Abstract

**Background:**

The occurrence of lupus nephritis is primarily caused by the dysfunction of the autoimmune system, leading to the deposition of immune complexes (ICs) in the kidneys and associated inflammatory responses. Lymphocyte-related parameters, including the platelet to lymphocyte ratio (PLR), neutrophil to lymphocyte ratio (NLR), and monocyte to lymphocyte ratio (MLR), have been confirmed in recent years as important novel indicators for several inflammatory diseases. However, it remains unclear whether lymphocyte-related parameters can serve as prognostic indicators for lupus nephritis (LN).

**Methods:**

This study included a total of 143 LN patients, who were divided into several groups based on the optimal cutoff values of lymphocyte-related parameters. The primary endpoint was poor renal prognosis, and the patients’ prognosis was monitored through follow-up, recording the time at which patients reached the study endpoint. The predictive effect was evaluated using the area under the receiver operating characteristic curve (AUROC), Kaplan-Meier (K-M) curves, and Cox proportional hazards analysis.

**Results:**

Compared with the healthy control group, the PLR, NLR, and MLR levels in the LN group were significantly higher (P < 0.05). Kaplan-Meier survival analysis showed that patients with high PLR, NLR, and MLR had poorer prognosis (P < 0.05). Univariate Cox regression analysis indicated that PLR (*HR* 1.002, 95% CI 1.000-1.004, P = 0.05) and NLR (*HR* 1.081, 95% CI 1.031-1.134, P = 0.001) were associated with kidney progression. Multivariate Cox regression analysis showed that only MLR (*HR* 5.861, 95% CI 1.515-22.665, P = 0.010) was an independent risk factor affecting the renal prognosis of LN patients, whereas PLR and NLR were not. Based on the cutoff value of MLR, patients were divided into two groups. In terms of general data, the high MLR group had a significantly higher mean arterial pressure compared to the low MLR group (P = 0.002). In terms of laboratory tests, the high MLR group had a significantly lower eGFR compared to the low MLR group (P = 0.001). In terms of renal pathology, the high MLR group showed statistically significant differences compared to the low MLR group in AI index, CI index, capillary endothelial cell proliferation, cellular/fibrous crescent formation, and interstitial inflammatory cell infiltration (P < 0.05).

**Conclusion:**

MLR may serve as an independent risk factor for poor renal prognosis in SLE patients.

## 1 Introduction

Systemic lupus erythematosus (SLE) is a typical autoimmune disease that affects multiple organs, particularly prevalent in women of childbearing age. Its clinical manifestations are diverse and can involve various organs and systems, including the kidneys, skin, and joints. Lupus nephritis (LN) is one of the most severe target organ damages in SLE, and it is also a major cause of poor prognosis in SLE patients, leading to end-stage renal disease (ESRD). Despite the gradual standardization of LN diagnosis and treatment with the introduction of LN guidelines in recent years, a portion of patients still experience disease progression. Within 10 years of the initial diagnosis of SLE, 5-20% of LN patients will progress to ESRD. Additionally, since 2000, the proportion of LN patients requiring renal replacement therapy has remained unchanged, and studies have shown an increasing trend in the proportion of LN patients requiring such therapy in recent years (1, 2). Currently, the diagnosis and treatment of LN remain challenging. The assessment of the degree of LN damage still relies on kidney biopsy; however, due to its invasive nature and potential complications (such as bleeding, infection, and perinephric hematoma), it is difficult to perform frequently during the treatment process. Therefore, relying solely on kidney biopsy to dynamically assess disease progression and treatment outcomes has certain limitations. To overcome these challenges, there is an urgent need to develop reliable, non-invasive biomarkers.

Lymphocyte-related parameters, including the platelet to lymphocyte ratio (PLR), neutrophil to lymphocyte ratio (NLR), and monocyte to lymphocyte ratio (MLR), have been confirmed in recent years as important novel indicators for several inflammatory diseases. In addition to serving as diagnostic tools, these lymphocyte-related ratios (PLR, NLR, MLR) also have significant value in the prognosis assessment of various diseases. Studies have shown that they can help predict disease progression and patient survival rates, including in coronary artery disease, various solid tumors, and rheumatoid arthritis (3–5). Apart from some systemic diseases, recent studies have also reported the association of PLR, NLR, and MLR with the phenotype and prognosis of kidney diseases, including chronic kidney disease, acute kidney injury, and rapidly progressive glomerulonephritis (6–8). However, previous studies have shown controversial results regarding the predictive value of PLR, NLR, and MLR for prognosis in LN patients, and more research is needed for confirmation. Therefore, the aim of this study is to evaluate the levels of PLR, NLR, and MLR in lupus nephritis patients and explore their relationship with renal prognosis in lupus nephritis.

## 2 Methods

### 2.1 Patient selection

Patients who visited the Department of Nephrology at the Second Hospital of Jilin University from January 2014 to October 2023, and who met the 1997 American College of Rheumatology (ACR) criteria for the diagnosis of systemic lupus erythematosus (SLE), and were diagnosed with lupus nephritis through kidney biopsy pathology, were included in this study. Relevant data at the time of kidney biopsy were collected. Simultaneously, 100 gender- and age-matched healthy volunteers from physical examination centers were recruited as the control group.

### 2.2 Inclusion criteria

Eligible patients were those diagnosed with SLE with complete renal pathology and laboratory data, meeting the following criteria:

1. Met the 1997 American College of Rheumatology (ACR) criteria for the diagnosis of systemic lupus erythematosus (SLE);
2. Diagnosed with lupus nephritis through kidney biopsy with clear pathological confirmation;
3. Complete laboratory and pathological data from the kidney biopsy.

### 2.3 Exclusion criteria

Patients with incomplete data or a history of blood transfusion, immunosuppressive therapy, infection, or other severe diseases were excluded if they met any of the following criteria:

1. Patients with incomplete follow-up data;
2. Patients who had a history of blood transfusion within 3 months prior to biopsy;
3. Patients who received glucocorticoids or other immunosuppressive treatments within 3 months prior to biopsy;
4. Patients in the acute or chronic inflammatory phase with a body temperature higher than 38.5°C, or with concurrent acute kidney injury;
5. Patients with severe comorbidities, such as chronic infectious diseases, diabetic nephropathy, hypertensive nephropathy, malignant tumors, lymphoproliferative disorders, other autoimmune diseases, or hematologic disorders.

Based on the inclusion and exclusion criteria, a total of 143 patients were enrolled in the study, including 18 male patients and 125 female patients, with a male-to-female ratio of 1:6.94. The median age was 36 years (range 20–50).

### 2.4 Clinical and pathological data collection

1. General Data: Includes gender, age, systolic blood pressure, diastolic blood pressure, height, and weight;
2. Laboratory Data: Absolute lymphocyte count, absolute monocyte count, absolute neutrophil count, platelet count, serum albumin, serum uric acid, serum creatinine, 24-hour urine protein quantification, erythrocyte sedimentation rate, complement C3, complement C4, anti-double-stranded DNA antibodies, and SLEDAI score;
3. Pathological Data: Pathological type of LN diagnosed through kidney biopsy at our hospital, results from light microscopy, immunofluorescence, and electron microscopy, as well as AI and CI indices.

### 2.5 Treatment and renal endpoint

Treatment was primarily based on the most recent KDIGO and EULAR guidelines (9–12)for the year of the kidney biopsy, with the final treatment plan determined by the attending physician in consultation with the patient. Treatment modalities mainly included glucocorticoids, antimalarials, immunosuppressive agents, and biologic agents.

Patient prognosis was monitored through telephone follow-ups, and the time when patients reached the study endpoint was recorded. The follow-up period extended until the patient’s death, loss to follow-up, or the study’s cutoff date, October 31, 2024. The primary endpoint was poor renal prognosis, defined as an eGFR < 60 ml/min, a ≥20% decline in eGFR from baseline, initiation of renal replacement therapy, or death.

### 2.6 Definition and predictive value of platelet-related parameters

PLR is calculated by dividing the absolute platelet count by the absolute lymphocyte count. The ratio of the absolute neutrophil count to the absolute lymphocyte count is the NLR. The ratio of the absolute monocyte count to the absolute lymphocyte count is the MLR.

### 2.7 Statistical analysis

Data was analyzed using R and SPSS software. A P value < 0.05 was considered statistically significant. For quantitative data, continuous variables with a normal distribution are expressed as mean ± standard deviation (X ± SD), and continuous variables with a non-normal distribution are expressed as median and interquartile range (M (P25, P75)). The Mann-Whitney U test was used for inter-group comparisons. Categorical data are expressed as frequencies and percentages, with inter-group comparisons conducted using the chi-square test, rank-sum test, and Fisher’s exact test. Receiver operating characteristic (ROC) curves were plotted to evaluate the predictive ability of each parameter for disease activity and poor renal prognosis, and to determine the optimal cutoff values for the ratios. Kaplan-Meier survival curves were generated to assess the value of each ratio in predicting renal survival. Multivariate analysis was performed using the Cox proportional hazards model.

## 3 Results

### 3.1 Comparison of PLR, NLR, and MLR values between LN patients and control group

**Figure 1:**
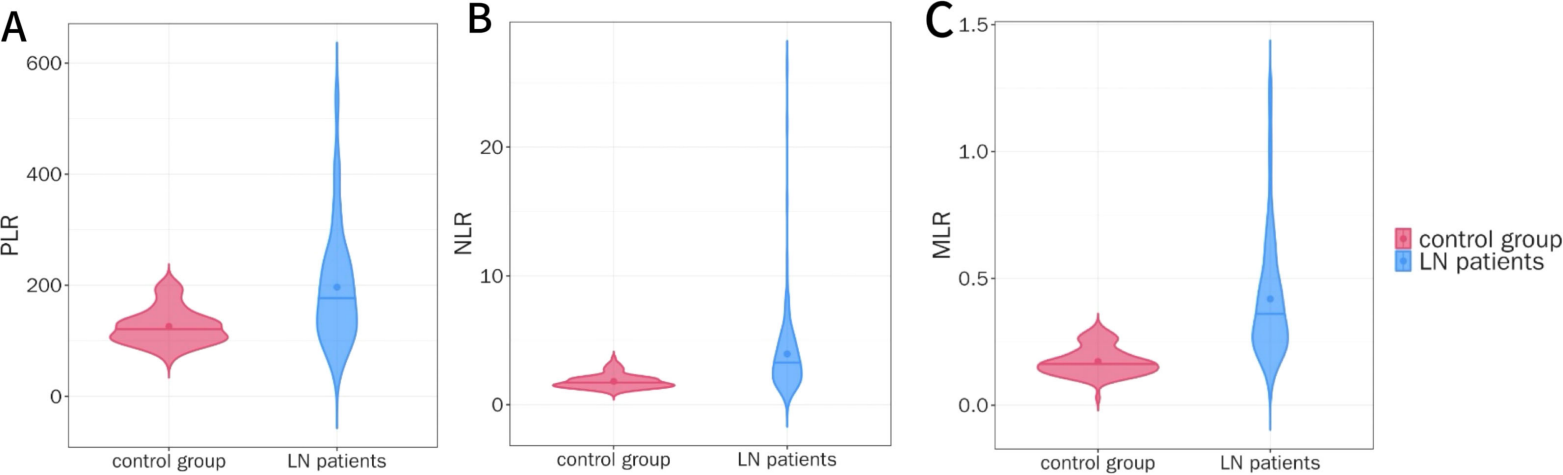
Comparison of PLR, NLR, and MLR Levels between the Control Group and the LN Group. **(A)** Comparison of PLR levels between LN patients and control group. **(B)** Comparison of NLR levels between LN patients and control group. **(C)** Comparison of MLR levels between LN patients and control group.

After strict inclusion and exclusion criteria, a total of 143 patients were included in this study. Compared with the healthy control group, the PLR, NLR, and MLR levels in the LN group were significantly higher (median 180.00 vs 123.16, 3.23 vs 1.66, 0.34 vs 0.17, P < 0.001), as shown in **Figures 1A–C**.

### 3.2 Predictive value of lymphocyte-related parameters for renal prognosis in LN patients

**Figure 2:**
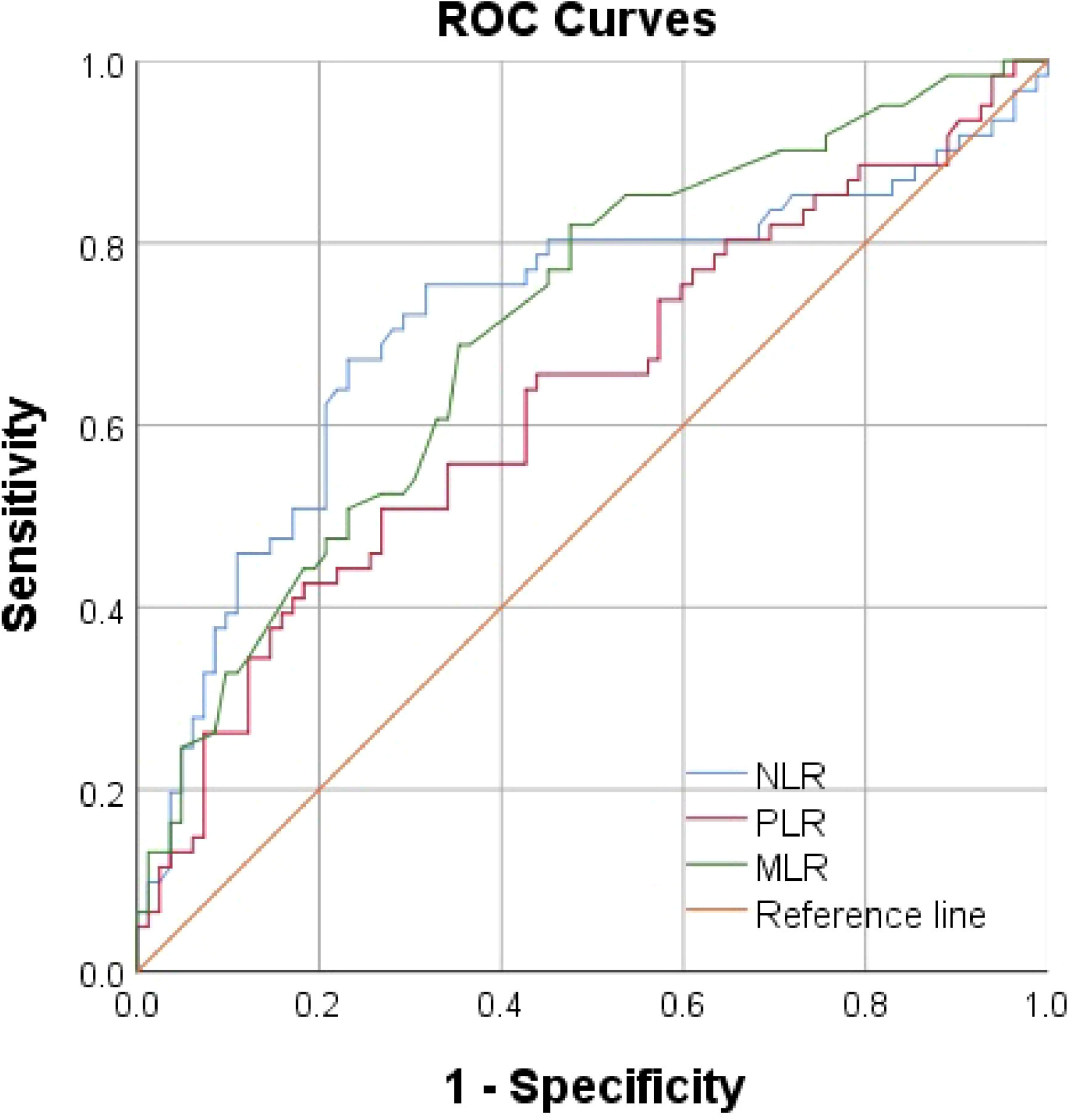
ROC Curves of PLR, NLR, and MLR in Predicting Poor Renal Prognosis in LN Patients.

**Figure 3:**
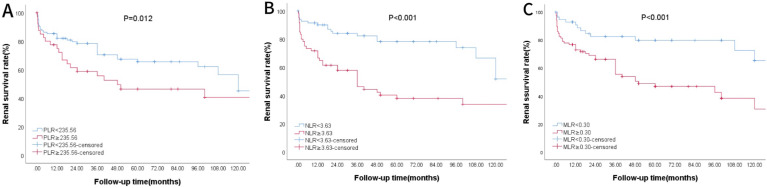
K-M Survival Curve of Patients Grouped by PLR, NLR and MLR. **(A)** Patients were divided by PLR. **(B)** Patients were divided by NLR. **(C)** Patients were divided by MLR.

Using AUROC to identify the prognostic value of lymphocyte-related parameters. Three main parameters (PLR, NLR, and MLR) were compared, all of which appeared associated with poor renal outcomes in lupus nephritis patients. The predictive ability of MLR was found to be superior to PLR and NLR (**Figure 2**, **Table 1**). As of the follow-up endpoint of this study (October 31, 2024), the median follow-up time was 36 months (range 10–70 months), and a total of 61 patients experienced outcome events (42.66%). There was a significant difference in renal survival rates among patients with different levels of PLR, NLR, and MLR (P < 0.05, **Figures 3**). The renal survival rates for the high and low MLR groups were 47/86 (54.65%) and 14/57 (24.56%), respectively. For the high and low NLR groups, the rates were 41/60 (68.33%) and 20/83 (24.10%), respectively (**Figures 3B, C**). Additionally, this study found that the clinical outcomes of patients in the high PLR group were generally worse than those in the low PLR group (P < 0.05, **Figure 3A**).

**Table 1:** Receiver operating characteristic (ROC) curve.

Variable	AUC (95%CI)	P value	Cutoff value	Sensitivity	Specificity
PLR	0.63 (0.54-0.73)	0.008^*^	235.56	42.62%	81.71%
NLR	0.72 (0.63-0.81)	<0.001^**^	3.63	67.21%	76.83%
MLR	0.71 (0.63-0.79)	<0.001^**^	0.30	81.97%	52.44%

*P < 0.05, the difference is statistically significant; **P < 0.001, the difference is statistically significant.

### 3.3 Cox regression analysis of the impact of MLR on the prognosis of LN patients

The renal endpoint of this study was eGFR < 60 ml/min, a 20% reduction in eGFR from baseline, or death. Through univariate Cox regression analysis, we found that PLR, NLR, and MLR levels were associated with the progression of LN (PLR:P=0.05; NLR:P=0.001; MLR:P < 0.001; **Table 2**). Additionally, the HR for MLR (MLR: HR7.999, 95%CI3.362-19.033) was higher than that for PLR and NLR (PLR: HR1.002, 95%CI1.000-1.004; NLR: HR1.081, 95%CI1.031-1.134). Further evaluation using a multivariate Cox regression model, which included general data, laboratory results, and pathological characteristics, showed that after adjusting for related factors, only MLR was an independent risk factor for the prognosis of LN patients (P=0.010), while PLR and NLR were not (**Table 2**).

**Table 2:** Cox regression analysis of PLR, NLR, MLR, and the prognosis of LN.

Parameter	HR (95%CI)			
	Model 0	Model 1	Model 2	Model 3
PLR	1.002 (1.000,1.004) 0.050^*^	1.002 (0.999,1.004) 0.182	1.002 (0.999,1.004) 0.212	1.001 (0.998,1.004) 0.479
NLR	1.081 (1.031,1.134) 0.001^*^	1.087 (1.032,1.144) 0.002^*^	1.058 (0.989,1.131) 0.099	1.061 (0.979,1.151) 0.150
MLR	7.999 (3.362,19.033) <0.001^**^	7.301 (2.943,18.114) <0.001^**^	4.397 (1.407,13.741) 0.011^*^	5.861 (1.515,22.665) 0.010^*^

*P < 0.05, the difference is statistically significant; **P < 0.001, the difference is statistically significant.

Model 0: Unadjusted for other influencing factors.

Model 1: Gender; Age; Mean Arterial Pressure; BMI

Model 2: Model 1 + Albumin; Uric Acid; eGFR; 24h Urine Protein Quantification; SLEDAI Score; ESR; Complement C3; Complement C4; Anti-dsDNA Antibodies

Model 3: Model 2 + Capillary Endothelial Cell Proliferation; Cellular or Fibrocellular Crescents; Nuclear Fragmentation and Necrosis; Hyaline or Transparent Thrombi; Glomerular Leukocyte Infiltration; Interstitial Inflammatory Cell Infiltration; Glomerulosclerosis; Tubular Atrophy; Fibrous Crescents; Interstitial Fibrosis

### 3.4 Comparison of LN patients in the low MLR group and high MLR group

From the above statistical results, it is evident that MLR is the most important prognostic indicator among all lymphocyte-related parameters. Therefore, we divided the patients into two groups based on the cutoff value of MLR and compared their general data, laboratory results, and pathological characteristics. The cutoff value for MLR was 0.30, which is the optimal threshold for distinguishing whether the renal prognosis of LN patients is poor (sensitivity 81.97%, specificity 52.44%). A total of 57 patients were classified into the low MLR group, and 86 patients were classified into the high MLR group.

### 3.5 Comparison of general data and laboratory results between the low MLR group and high MLR group

In terms of general data, the high MLR group had a higher mean arterial pressure compared to the low MLR group, with a statistically significant difference (P = 0.002). In terms of laboratory tests, the high MLR group had a significantly lower eGFR compared to the low MLR group, with a statistically significant difference (P = 0.001). No statistically significant differences were observed between the two groups in other laboratory tests, as shown in **Table 3**.

**Table 3:** Comparison of general data and laboratory results between the low MLR group and high MLR group.

Parameter	Low MLR group	High MLR group	P value
Number (%)	57.00 (39.86)	86.00 (60.14)	
Age (years)	33.00 (25.50,47.00)	38.00 (27.00,52.00)	0.374
Gender (Male, %)	9.00 (15.78)	9.00 (10.47)	0.347
Mean Arterial Pressure (mmHg)	96.00 (90.99,105.83)	100.33 (93.33,112.00)	0.002^*^
BMI (kg/m²)	22.19 (19.80,24.26)	22.62 (20.69,25.47)	0.111
Albumin (g/L)	28.50 (24.10,33.25)	26.15 (21.88,21.60)	0.200
Uric Acid (µmol/L)	369.00 (311.50,488.50)	392.00 (333.00,484.00)	0.373
eGFR (ml/min)	105.51 (81.13,121.35)	61.29 (37.50,101.80)	<0.001^*^
24-hour Urine Protein Quantification (g/24h)	3.35 (1.20,6.06)	3.47 (1.66,5.99)	0.851
SLEDAI Score (points)	17.53 ± 5.88	18.76 ± 5.75	0.718
ESR (mm/h)	47.05 ± 27.94	52.45 ± 31.08	0.318
C3(mg/dl)	32.30 (21.20,58.85)	32.20 (21.15,45.10)	0.553
C4(mg/dl)	7.71(3.35,14.70)	6.15(3.55,12.65)	0.704
Anti-dsDNA Antibodies (IU/ml)	649.00(100.00,1041.50)	478.50(100.00,900.00)	0.200

*P < 0.001, the difference is statistically significant.

### 3.6 Comparison of pathological data between the low MLR group and high MLR group

In terms of renal pathology, the high MLR group showed statistically significant differences compared to the low MLR group in AI, CI, capillary endothelial cell proliferation, cellular crescent/fibrocellular crescent formation, and interstitial inflammatory cell infiltration (P < 0.05). However, no statistically significant differences were observed between the two groups in nuclear fragmentation and necrosis, hyaline or transparent thrombi, glomerular leukocyte infiltration, glomerulosclerosis, tubular atrophy, fibrous crescent formation, and interstitial fibrosis (P > 0.05), as shown in **Table 4**.

**Table 4:** Comparison of pathological data between the low MLR group and high MLR group.

Characteristic	Low MLR group	High MLR group	P value
AI (points)	4.00 (2.00,7.50)	7.00 (5.00,10.00)	<0.001^**^
CI (points)	2.00 (1.00,3.00)	3.00 (2.00,4.00)	0.015^*^
Capillary Endothelial Cell Proliferation (cases, %)	0 points:15.00 (26.32)	0 points:14.00 (16.28)	0.007^*^
	1 points:16.00 (28.07)	1 points:14.00 (16.28)	
	2 points:11.00 (19.30)	2 points:10.00 (11.63)	
	3 points:15.00 (26.32)	3 points:48.00 (55.81)	
Cellular Crescent/Fibrocellular Crescent (cases, %)	0 points:40.00 (70.18)	0 points:38.00 (44.19)	0.004^*^
	2 points:17.00 (29.82)	2 points:41.00 (47.67)	
	4 points:0 (0.00)	4 points:6.00 (7.00)	
	6 points:0 (0.00)	6 points:1.00 (1.16)	
Nuclear Fragmentation and Necrosis (cases, %)	0 points:38.00 (66.67)	0 points:46.00 (53.49)	0.080
	2 points:18.00 (31.58)	2 points:40.00 (46.51)	
	4 points:0 (0.00)	4 points:0 (0.00)	
	6 points:1.00 (1.75)	6 points:0 (0.00)	
Hyaline or Transparent Thrombi (cases, %)	0 points:34.00 (59.65)	0 points:42.00 (48.84)	0.067
	1 points:21.00 (36.84)	1 points:38.00 (44.19)	
	2 points:2.00 (3.51)	2 points:1.00 (1.16)	
	3 points:0 (0.00)	3 points:5.00 (5.81)	
Glomerular Leukocyte Infiltration (cases, %)	0 points:48.00 (84.21)	0 points:58.00 (67.44)	0.072
	1 points:8.00 (14.04)	1 points:25.00 (29.07)	
	2 points:0 (0.00)	2 points:0 (0.00)	
	3 points:1.00 (1.75)	3 points:3.00 (3.49)	
Interstitial Inflammatory Cell Infiltration (cases, %)	0 points:14.00 (24.56)	0 points:10.00 (11.63)	0.040^*^
	1 points:31.00 (54.39)	1 points:40.00 (46.51)	
	2 points:4.00 (7.02)	2 points:11.00 (12.79)	
	3 points:8.00 (14.04)	3 points:25.00 (29.07)	
Glomerulosclerosis (cases, %)	0 points:21.00 (36.84)	0 points:24.00 (27.91)	0.061
	1 points:34.00 (59.65)	1 points:49.00 (57.00)	
	2 points:2.00 (3.51)	2 points:9.00 (10.47)	
	3 points:0 (0.00)	3 points:4.00 (4.65)	
Tubular Atrophy (cases, %)	0 points:21.00 (36.84)	0 points:24.00 (27.91)	0.168
	1 points:34.00 (59.65)	1 points:49.00 (57.00)	
	2 points:2.00 (3.5)	2 points:9.00 (10.47)	
	3 points:0 (0.00)	3 points:4.00 (4.65)	
Fibrous Crescent (cases, %)	0 points:22.00 (38.60)	0 points:20.00 (23.26)	0.277
	1 points:29.00 (50.88)	1 points:49.00 (57.00)	
	2 points:4.00 (7.02)	2 points:13.00 (15.12)	
	3 points:2.00 (3.51)	3 points:4.00 (4.65)	
Interstitial Fibrosis (cases, %)	0 points:48.00 (84.21)	0 points:66.00 (76.74)	0.363
	1 points:9.00 (15.79)	1 points:19.00 (22.09)	
	2 points:0 (0.00)	2 points:0 (0.00)	
	3 points:0 (0.00)	3 points:0 (0.00)	

*P < 0.05, the difference is statistically significant; **P < 0.001, the difference is statistically significant.

## 4 Discussion

Our results show that compared with the healthy control group, LN patients had significantly higher levels of PLR, NLR, and MLR (P < 0.05). Kaplan-Meier curves, without considering covariates, revealed that PLR, NLR, and MLR were closely associated with renal outcomes in LN patients (P < 0.05), suggesting that they may serve as potential predictive factors. ROC curve analysis further indicated that NLR and MLR had better predictive ability than PLR, with NLR and MLR showing similar predictive value. After adjusting for general data, laboratory tests, and renal pathology characteristics in multivariate Cox regression analysis, MLR was found to be a better marker for predicting poor renal outcomes than PLR and NLR, indicating that MLR is the only lymphocyte-related parameter that can serve as an independent risk factor.

Platelet activation is triggered by both innate and adaptive immune stimuli. Once activated, platelets release immune-active molecules and interact with immune cells, promoting inflammation and thrombosis, which leads to organ damage in SLE (13). Experimental studies show that anti-platelet aggregation treatment reduces renal inflammation, complement deposition, anti-cardiolipin antibody levels, and thromboxane B2 levels in MRL/lpr mice, suggesting that platelet activation plays a pathogenic role in LN (14). A cross-sectional study from the United States further confirmed this hypothesis. In SLE, circulating immune complexes activate complement, and the generated complement cleavage products can bind to platelets to form PC4d. They found that PC4d is a biomarker of increased platelet activity (15). Additionally, platelets can shed membrane vesicles, known as microparticles or microvesicles, which carry and spread mitochondrial antigens, complement activation products, and other molecules, playing a role in forming immune complexes and mediating immune damage (16). Finally, platelets and their exosomes are the main source of TGF-β in circulation. Platelets and their exosomes promote renal interstitial fibrosis by releasing TGF-β, thereby exacerbating kidney damage (17). Several studies in adolescent SLE patients have demonstrated that those with elevated PLR are more prone to coagulation abnormalities and cutaneous rash (18). These findings suggest that PLR may serve as a predictive biomarker for both SLE disease activity and rash manifestation. Currently, there is no research proving the association between PLR and renal prognosis in LN patients.

The presence of neutrophil infiltration in renal biopsy specimens suggests that inflammatory peptides and cytokines derived from circulating neutrophils may be involved in the pathogenesis of LN. Dysregulated neutrophil activation plays an important role in the onset and progression of SLE, where impaired apoptosis and NET formation expose proteins and DNA with post-translational modifications, triggering adaptive immune responses (such as interferon release and antibody production), and causing tissue damage either directly or by activating adjacent cells (19). NETs, composed of nuclear and granular components released from activated neutrophil membranes, play a key role in the balance between NET production and clearance in SLE and other autoimmune diseases. Studies show that impaired NET clearance is associated with disease activity in SLE patients. Notably, patients with reduced NET clearance have lower levels of circulating complement components C3 and C4 (20). In SLE, dysregulated apoptosis of neutrophils leads to an increased apoptotic load, which is associated with the production of antinuclear autoantibodies (19). Regarding the relationship between NLR and renal prognosis in LN patients, our study suggests that NLR has some predictive value for poor renal outcomes, but NLR does not appear to be an independent risk factor for renal prognosis. Zhou et al. reported similar findings, where their univariate Cox regression analysis indicated that NLR was a risk factor for renal prognosis in LN patients, but after adjusting for general data and laboratory results, NLR was not an independent risk factor for renal prognosis in LN patients (21). However, Chen et al. obtained different results. They included 122 LN patients and divided them into low, medium, and high NLR groups. They combined the medium and low groups in multivariate Cox regression and found that high NLR levels were an independent risk factor for poor prognosis in LN patients (22). The inconsistency in these results may be due to geographic variations in the samples included and the small sample size, or it may be related to their failure to exclude the effects of glucocorticoids and immunosuppressants.

Lymphocytes are closely associated with adaptive immunity. Lymphopenia, a common complication in SLE, is linked to multiple factors including lymphocytotoxic antibodies, excessive apoptosis, increased susceptibility to complement-mediated lysis, and suppressed lymphopoiesis. Notably, lymphopenia correlates with disease activity and elevated risk of organ damage (23). As early as 2019, a study from Denmark found that lymphopenia was an independent risk factor for the first onset of proteinuria in SLE patients (24). The study found that compared with healthy controls, LN patients had significantly reduced lymphocyte counts, mainly affecting the CD4 cell subset. Renal pathology classification in LN patients was mainly associated with changes in CD4 lymphocytes, with peripheral CD4 cell reduction observed in patients with active and proliferative lesions (25). Additionally, Abraham et al. found that a higher density of B cells at the time of renal biopsy was associated with lower chronic renal tubulointerstitial inflammation scores and better prognosis, suggesting that B cells may have a previously unrecognized protective role in the kidneys (26). Recently, several studies focused on LN microenvironment and used single-cell RNA-seq technique to reveal the role of MLR in LN progression. Chemokine receptors CXCR4 and CX3CR1 were broadly expressed in LN kidney, indicating the potential therapy target of LN on cell trafficking (27). Chen et al. observed the enrichment of CD163 dendritic cells (DC3s) in LN kidneys, which exhibited a positive correlation with the severity of LN. The crosstalk involving DC3s, T cells and tubular epithelial cells within LN kidneys may play a significant role in elucidating disease progression mechanisms and could provide potential therapeutic targets for clinical intervention (28). Single cell sequencing analysis also revealed the overactivation of granzyme K CD8 T cells in the kidney of patients with LN and associated extrafollicular B cell response, which may suggest a potential new intervention target for LN. Lymphopenia seems to be not only a laboratory result of disease activity in SLE patients, but also possibly related to renal involvement in SLE patients. This study found that MLR is an independent risk factor for poor renal prognosis in LN patients. The high MLR group showed higher mean arterial pressure and lower eGFR. In terms of renal pathology, the high MLR group had significantly higher AI and CI indices, and the incidence of capillary endothelial cell proliferation, cellular crescent/fibrocellular crescent formation, and interstitial inflammatory cell infiltration was significantly higher than in the low MLR group. One study found that in SLE patients, MLR was positively correlated with C-reactive protein and negatively correlated with IgM (29). Additionally, Liu et al. reported that compared with healthy controls, MLR levels were significantly increased in LN patients without infection (30), which is consistent with our results. However, to our knowledge, there is currently no research on the relationship between MLR and renal prognosis in LN patients, which is a key difference between our study and previous research. A retrospective study showed that for end-stage kidney disease patients requiring renal replacement therapy for 6 months, MLR at admission had strong predictive ability for all-cause 30-day mortality. Elevated MLR was also associated with longer hospital stays and more dialysis sessions per patient (31). Similar results were found in studies of acute kidney injury patients, where higher baseline MLR was identified as an independent risk factor for predicting 30-day and 90-day mortality. Early increases in MLR were associated with higher 30-day mortality (32). Furthermore, Zhang et al. reported the predictive value of MLR in primary membranous nephropathy, finding that higher MLR was associated with poor renal outcomes (33). Therefore, MLR may be a new tool for predicting poor renal outcomes, and larger-scale studies are needed to confirm our hypothesis.

In type II and IV LN, immune complex deposition in capillaries has been observed, and monocytes expressing FcγRIII (also known as CD16) are considered to be associated with the pathogenesis of this disease. In type IV LN, CD16+ monocytes are found at the sites of capillary immune complex deposition, which is related to the expression of the endothelial cell CX3C chemokine ligand 1 (CX3CL1), which may promote the aggregation of these monocytes, suggesting an important role of monocytes in the pathogenesis of LN (34). Macrophages, derived from monocytes, have shown heterogeneity, with both pro-inflammatory and anti-inflammatory functions. In lupus nephritis, these two functions are imbalanced, leading to chronic inflammation, fibrosis, and renal dysfunction. Monocytes mediate varying immune responses through circulating immune complexes and release proinflammatory mediators, triggering microvascular endothelial injury and increased permeability. This pathogenic cascade accelerates the development of SLE complications, particularly lupus nephritis (35). Therefore, targeted macrophage therapy is a potential new treatment approach for LN (36).

The innovation of this study lies in two aspects: First, PLR, NLR, and MLR can be calculated from routine blood cell counts, offering lower cost and easier accessibility compared to other biomarkers predicting disease activity and prognosis in LN patients. Second, considering these hematological parameters are susceptible to various medications and physicochemical factors, this study—unlike others—excluded patients who had received glucocorticoid or other immunosuppressive treatments within three months prior to renal biopsy. This research also has several potential limitations. Firstly, as a single-center retrospective study with an insufficient sample size, future investigations should adopt multicenter prospective designs with larger cohorts for statistical evaluation and validation. Secondly, although we excluded interference from glucocorticoids and immunosuppressants, PLR, NLR, and MLR may still be influenced by other drugs and physicochemical factors, potentially introducing bias. Finally, data of absolute values for macrophages and long-term follow-up with survival data collection remains necessary to further elucidate the relationship between MLR and LN prognosis.

In conclusion, we have discussed the pathogenic roles of lymphocytes, platelets, neutrophils, and monocytes in LN. However, the increase in PLR, NLR, and MLR does not necessarily reflect an absolute increase in platelet, neutrophil, and monocyte counts, or an absolute decrease in lymphocyte counts. In fact, in SLE patients, the incidence of thrombocytopenia, neutropenia, and lymphopenia is 10-40%, 20-40%, and 15-82%, respectively (37, 38). Although the pathogenesis of lupus nephritis is still not fully understood, current research suggests that autoimmune inflammatory responses are an important pathophysiological mechanism for multi-organ and tissue damage, particularly kidney injury. PLR, NLR, and MLR reflect the ratios of platelets, neutrophils, monocytes to lymphocytes, and as a combination of these cell counts, they have lower sensitivity to physical, biochemical, or physiological factors compared to individual indicators, making them more valuable in predicting inflammation. Therefore, they were selected as testing indicators in our study. However, further studies are needed to clarify whether the ratio between specific cell types provides higher diagnostic accuracy for LN than individual cell populations.

## Data Availability

The raw data supporting the conclusions of this article will be made available by the authors, without undue reservation.
